# Serological and molecular prevalence study of bluetongue virus in small domestic ruminants in Morocco

**DOI:** 10.1038/s41598-022-24067-y

**Published:** 2022-11-14

**Authors:** Soukaina Daif, Ikhlass El Berbri, Youssef Lhor, Ouafaa Fassi Fihri

**Affiliations:** 1 Microbiology, Immunology, and Infectious Diseases Unit, Department of Pathology and Veterinary Public Health, Institut Agronomique et Vétérinaire Hassan II, Rabat-Instituts, BP: 6202, Rabat, Morocco; 2 National Office of Food Safety (ONSSA), Rabat-Instituts, BP: 6202, Rabat, Morocco

**Keywords:** Molecular biology, Microbiology, Virology, Infectious diseases

## Abstract

Bluetongue is an arthropod-borne viral disease transmitted by *Culicoides* biting midges, affecting domestic and wild ruminants. The current study aims to assess the seroprevalence of the bluetongue virus (BTV) and confirm its active circulation among sheep and goats populations in Morocco, as well as study the risk factors associated with BTV infection. To this end, a total of 1651 samples were randomly collected from 1376 sheep and 275 goats in eight (out of 12) regions of the country between March 2018 and July 2021.These samples were primarily tested using competitive ELISA (c-ELISA). Subsequently, 65% of c-ELISA positives (n = 452) were analyzed by real-time reverse transcription-polymerase chain reaction (RT-qPCR). The results revealed an overall BTV seroprevalence in small ruminants in Morocco of 41.7%, including 42.6% in sheep and 37.5% in goats. The RT-qPCR results showed that the overall BTV viropositivity rate was 46.7%, including 48.1% in sheep and 41.8% in goats. These viro-serological rates varied significantly by age, sex, and breed of the tested animals, husbandry method, season, and geographic origin. This indicates that these parameters constitute risk factors for BTV transmission routes in Morocco. The findings also indicate that goats play a role as reservoirs in maintaining the BTV in Morocco. It appears from this study that bluetongue is endemic in Morocco. The environmental and climate conditions as well as the husbandry methods adopted in the country are particularly favorable for the virus transmission throughout the country.

## Introduction

Bluetongue is a viral disease of domestic and wild ruminants caused by the bluetongue virus (BTV) and transmitted primarily by *Culicoides* biting midges^1^. Bluetongue is a disease currently notifiable to the World Organization for Animal Health (WOAH). The clinical incidence varies according to the animal species and BTV serotype^2^. The repercussions are more severe in the sheep sector, with significant economic losses in flocks attributable to abortions, reduced production parameters and fertility rates, diagnostic and control measure costs, and trade restrictions^3^. Bluetongue virus is a small non-enveloped virus of the *orbivirus* genus within the *Reoviridae* family^4^, with ten double-stranded RNA linear segments encoding seven structural (VP1–VP7) and five non-structural proteins (NS1, NS2, NS3/NS3A, NS4, NS5)^5^. To date, 27 serotypes were described worldwide, including BTV-25 detected in Switzerland in 2007^6^, BTV-26 identified in Kuwait in 2010^7^, and BTV-27 isolated from goats in Corsica in 2014^8^. In addition, novel putative serotypes have been recently identified. BTV-28 (grouping BTV-28/1537/14 with SPvvv/03) and BTV-29 (SPvvv/02) isolated from sheeppox vaccine, BTV-30 which combine BTV-XJ1407 (China) and BTV-MNG2/2016 (Mongolia), BTV-31 (V196/XJ/2014) isolated in China, BTV-32 (BTV-X ITL2015) in Italy, BTV-33 (BTV-MNG3/2016) in Mongolia, BTV-34 (BTV-Y TUN2017) in Tunisia, BTV-35 (BTV-MNG1/2018) in Mongolia, and BTV-36 (BTV-36-CH2019) in Switzerland. Novel BTV strains were also detected in alpacas in South Africa but have not been genotyped^9–16^. Laboratory diagnosis of the disease is based on serological and molecular techniques for serogrouping and serotyping of BTV^17^. The VP2, the most variable structural protein that triggers neutralizing antibodies production, determines BTV serotypes^18^. The VP7 protein is relatively conserved and serves as the main immunogenic serogroup-reactive antigen of BTV^19^. The detection of Anti-VP7 antibodies is the primary target of the BTV ELISA serological assays^1^.

Bluetongue virus have been identified in many tropical, subtropical, and temperate areas between 40° North and 35° South latitudes^20^, where the climatic factors are favorable to the activity of *Culicoides* spp. In Morocco, bluetongue was first reported in 1956 in the southern zone of Larache and west of Arbaoua (in the norwestof the country). At this time, a limited circulation of BTV-10 had been confirmed^21^. The BTV reemerged again in 2004 in the Larache province, with the BTV-4 serotype^22^. The epidemic caused 230 outbreaks in 14 provinces over the country with 1876 cases in the small ruminant population and an average mortality rate of 1.3%^22^. In September 2006, a new serotype of BTV, BTV-1, emerged in the east of Morocco^22^. A total of 505 outbreaks were reported in 19 provinces, with 2043 cases and an average mortality rate of 0.76%^22,23^. Since then, different outbreaks have been reported all over the country, despite the national vaccination programs carried out in and out the initial outbreaks. Hence, epidemiological surveillance surveys are especially important to evaluate the current situation of the disease and assess the risk factors leading to BT outbreaks. The current study was conducted to define the epidemiological situation of bluetongue disease through epidemiological, serological, and molecular surveys in small ruminants in different regions (8 out of 12) of Morocco. These surveys aimed to assess the seroprevalence of BTV at the national level using the competitive ELISA (c-ELISA) test, confirm the active circulation of the virus by real-time RT-PCR, and identify the risk factors related to BTV infection in the species studied.

## Materials and methods

### Study area

Morocco is situated in the subtropical zone of Northwest Africa and has sizeable small ruminant herds of nearly 21.6 million sheep and 6 million goats. The Moroccan climate is diverse, with Mediterranean influences in the north, oceanic in the west, continental inland, and Saharan in the south. The study covered eight (out of 12) Moroccan regions located at low to medium altitudes in Morocco's south, west, east, center, and north (Figs. 2, 5).

### Blood sample collection

Blood samples of 1651 small ruminants (1376 sheep and 275 goats) were collected randomly from 79 unvaccinated (against BTV) ruminant farms between March 2018 and July 2021. The sample size within each farm varied from 25 to 100%. The tested animals were of different breeds, including: Sardi, Timahdite, and crossbreed breeds of sheep and indigenous breed of goats. The Sardi and Tamhdite are the major indigenous Moroccan sheep breeds, given their large numbers and wide geographical distribution. Blood was collected in dry tubes (for sera) and EDTA tubes (for whole blood) for each animal.

### Ethics statement

The samples were collected by qualified veterinarians following standard sample collection techniques without injury or stress to the animals. The animal breeders were always informed of the objectives and the nature of the analysis. All animal procedures are in agreement with the Agronomy and Veterinary Institute Hassan II of Rabat and the Moroccan Ministry of Agriculture recommendations, which are in accordance with international ethical standards (European Union Directive 2010/63/EU) legislation and ARRIVE (Animal Research Reporting of In Vivo Experiments) guidelines.

### Competitive Enzyme-Linked Immunosorbent Assay (c-ELISA)

All the collected 1651 sera were tested for antibodies against BTV-VP7 using ID Screen^®^ bluetongue competition Kit (ID-Vet, France). The c-ELISA test was performed according to the manufacturer's instructions. The absorbance (optical density) of the ELISA test results was read at a wavelength of 450 nm. The percentage of inhibition was calculated using the following formula: % inhibition = [(OD sample/OD negative controls) × 100]. Serum samples with a percentage of inhibition lower than or equal to 35% were considered positive to BTV-VP7 antibodies, greater than 35% and less than or equal to 45% are considered doubtful, and higher than 45% are considered negative.

### Molecular assays

#### Viral RNA extraction

A total of 65% (n = 452) of ELISA-positive samples was randomly selected for molecular analysis by real-time RT-PCR.

Total RNA was extracted from the 452 EDTA blood samples using the MagMAX™ Viral RNA Isolation Kit (Applied Biosystems) as per the test's instructions.

#### Real-time reverse transcription-polymerase chain reaction (RT-qPCR)

BTV RNA was detected using the LSI VetMAX™ BTV NS3 All Genotypes kit (Applied Biosystems) according to the manufacturer’s instructions. This kit targets the segment 10 of the viral genome that encodes NS3 protein, and it is based on specific duplex detection of the BTV by a FAM™-NFQ-labeled TaqMan^®^ probe and the internal positive control by VIC™–TAMRA™.

The reverse transcription and amplification were performed on an Applied Biosystems 7500 Fast Real-Time PCR System using the thermal cycler program: 45 °C for 10 min, 95 °C for 10 min, followed by 40 cycles of 95 °C for 15 s and 60 °C for 45 s. Fluorescence was measured at the 60 °C-45 s step. The interpretation of the test results was done according to the manufacturer’s instructions. A sample is positive for BTV if there is a sigmoidal amplification curve in the FAM-NFQ channel and the Ct value is not higher than 40.

### Data and statistical analysis

The data were analyzed according to eight factors, including species, age, sex, breed of the animals, type of breeding, season, year of survey, and region of origin. The proportions of seroprevalences and viropositivity rates according to these factors were compared by calculating 95% confidence intervals with the Mantel–Haenszel Chi-Square test in OpenEpi© Software (2021).

## Results

### Competitive ELISA results

BTV-VP7 antibodies were detected in 689 of 1651 sera tested, hence an overall seroprevalence of **41.7%**. The BTV seroprevalence was high in sheep (**42.6%**; 586/1376) compared to goats (**37.5%**; 103/275). Statistical analysis showed that the difference in the seroprevalence between the two species was not significant (χ^2^ = 2.483, P ˃ 0.05).

### In sheep

**Figure 1:**
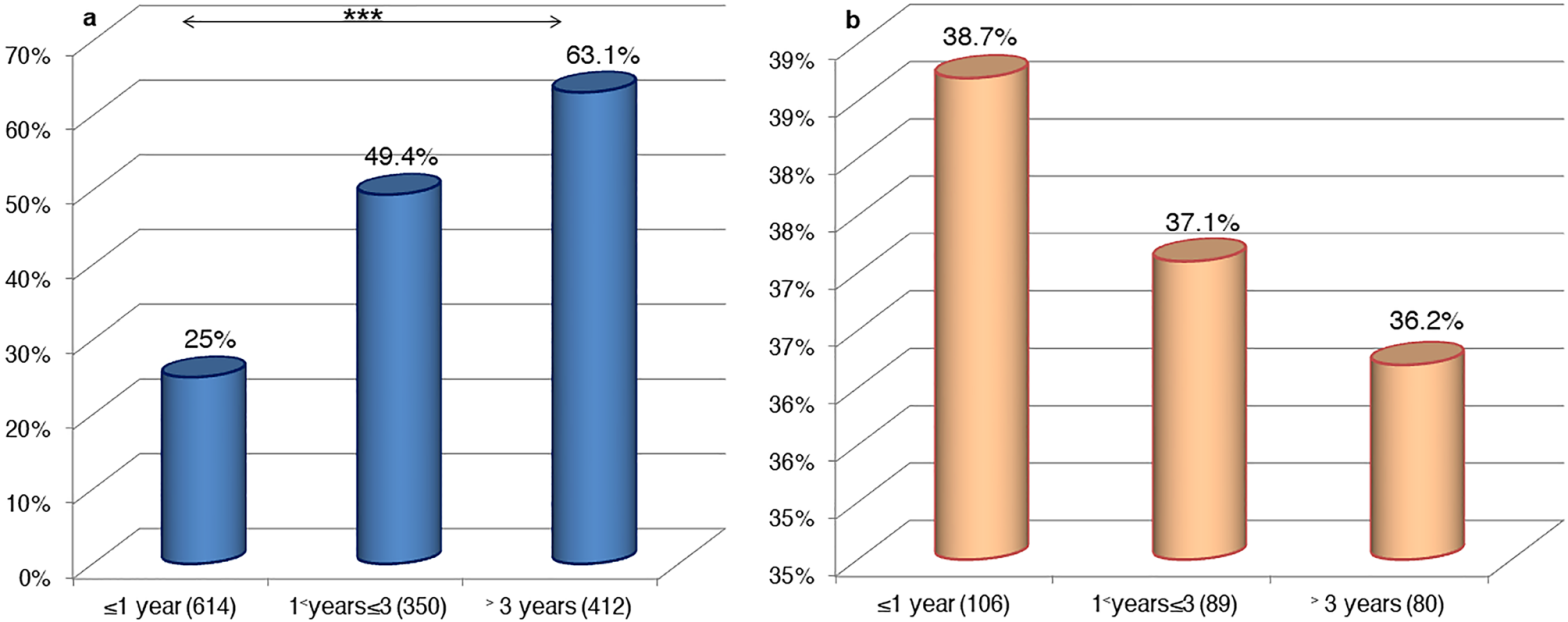
BTV antibody detection results by age in sheep and goats during March 2018–July 2021 in Morocco. (**a**) In sheep. (**b**) In goats (in brackets, the number of samples screened). The statistical significance between rates is indicated as ***P < 0.001.

**Figure 2:**
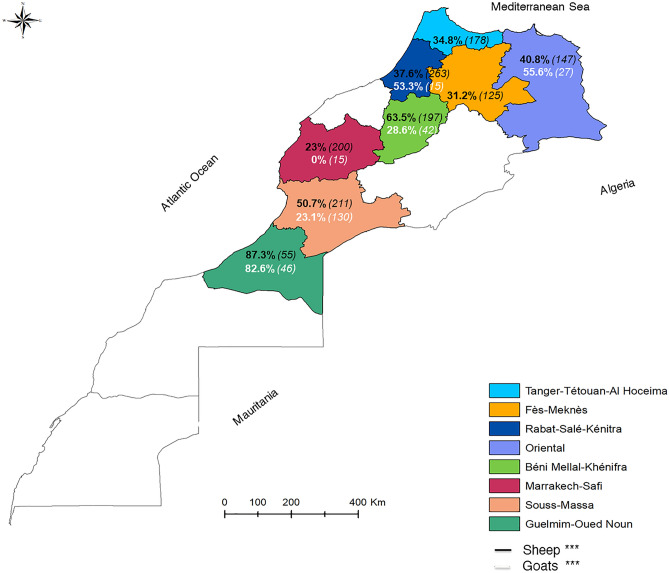
Bluetongue virus seroprevalences in sheep and goats during March 2018–July 2021 in Morocco. Black and white colors indicate BTV seroprevalence in sheep and goats, respectively (in brackets, the number of samples screened).The statistical significance between rates is indicated as ***P < 0.001.

The BTV seroprevalence varied significantly (P < 0.05) in sheep according to the age, sex, breed, and geographic origin of tested animals. Indeed, the oldest animals had higher seropositivity than the younger ones (χ^2^ = 156, P < 0.001) (Fig. 1a). Females were more frequently seropositive (**52%**; 455/875) than males (**26.1%**; 131/501) (χ^2^ = 87.08, P < 0.001). The Timahdite and Sardi breeds showed the highest seropositivity (**46.7%** (146/313) and **43%** (359/836), respectively), while the crossbreed sheep showed the lowest one (**35.7%**; 81/227) (χ^2^ = 6.577, P < 0.05). Furthermore, a highly significant variation in seroprevalences rates (χ^2^ = 130.9, P < 0.001) was observed by the geographic origin of animals, the region of Guelmim-Oued Noun was the one with the higher rate (**87.3%**; 48/55) (Fig. 2).

### In goats

As with sheep, a significant variation in seroprevalences rates (χ^2^ = 82.82, P < 0.001) was observed by the geographic origin of animals and the higher rate was observed in the Guelmim-Oued Noun region (**82.6%**; 38/46) (Fig. 2).

For goats, males (**46.6%**; 40/86) had significantly higher seroprevalence than females (**33.3%**; 63/189) (χ^2^ = 4.382, P < 0.05). However, the seropositivity decreased slightly and non-significantly with age (χ^2^ = 0.1228, P ˃ 0.05) (Fig. 1b). All goats surveyed were of an indigenous breed and exhibited a considerable seroprevalence (**37.5%**; 103/275).

### Variation in BTV seroprevalence in Moroccan flocks

The average BTV seroprevalence in the extensive farms was **49.4%** (522/1057), compared to **28.1%** (167/594) in intensive breeding (χ^2^ = 70.76, P < 0.001). In addition, the seroprevalence (**49.8%**; 347/697) in sheep from mixed livestock farms (sheep with goats and/or cattle farms) was significantly higher than that (**35.2%**; 239/679) in sheep from exclusively sheep farms (χ^2^ = 29.93, P < 0.001).

### Variation in BTV seroprevalence by year and month

**Figure 3:**
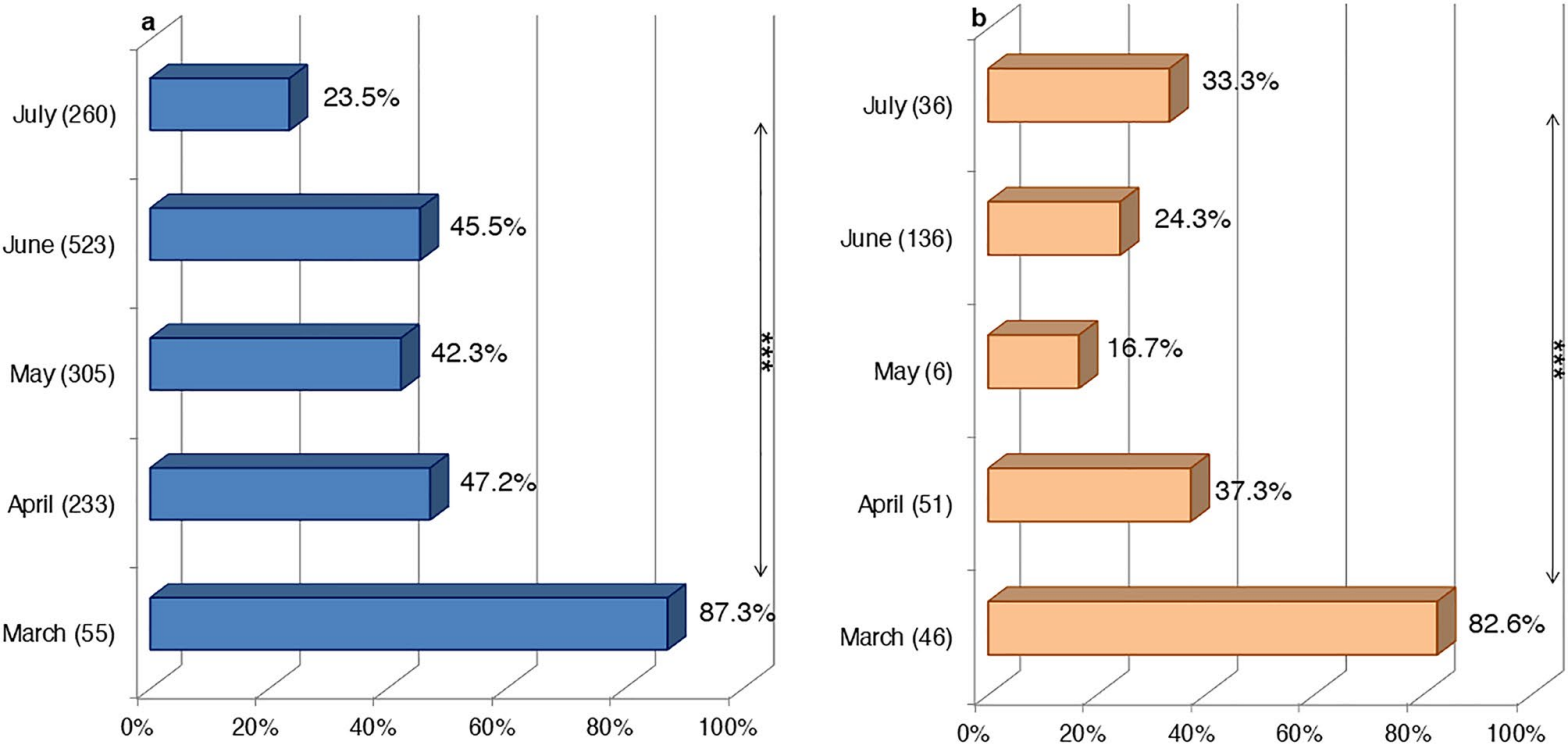
BTV seroprevalence distribution by month of sampling in sheep and goats during March 2018-July 2021 in Morocco. (**a**) In sheep. (**b**) In goats (in brackets, the number of samples screened). The statistical significance between rates is indicated as ***P < 0.001.

The samples were collected between March 2018 and July 2021. It is worth noting that the number of samples collected and their regions of origin were different over the four years. The results revealed that the overall BTV seroprevalence was **41.7%** (337/808) in 2018, **40.9%** (269/657) in 2019, **66.7%** (8/12) in 2020, and **43.1%** (75/174) in 2021. The variation in BTV seroprevalence by year was not statistically significant (χ^2^ = 3.371, P ˃ 0.05). According to the sampling month, March registered the highest average BTV seroprevalence in sheep and goats (**87.3% **(48/55) and **82.6%** (38/46), respectively) (Fig. 3a,b). The statistical analysis demonstrated a highly significant difference between BTV seroprevalence and sampling months in sheep and goats (χ^2^ = 87.68, P < 0.001; χ^2^ = 51.5, P < 0.001, respectively).

### RT-qPCR results

Out of 452 samples screened, the BTV viropositivity rate was **46.7%**. The virus was active in sheep and goats with a higher percentage in sheep (**48.1%** (168/349), and **41.8%** (43/103), respectively). However, the difference in rates by species was not statistically significant (χ^2^ = 1.305, P ˃ 0.05).

### In sheep

**Figure 4:**
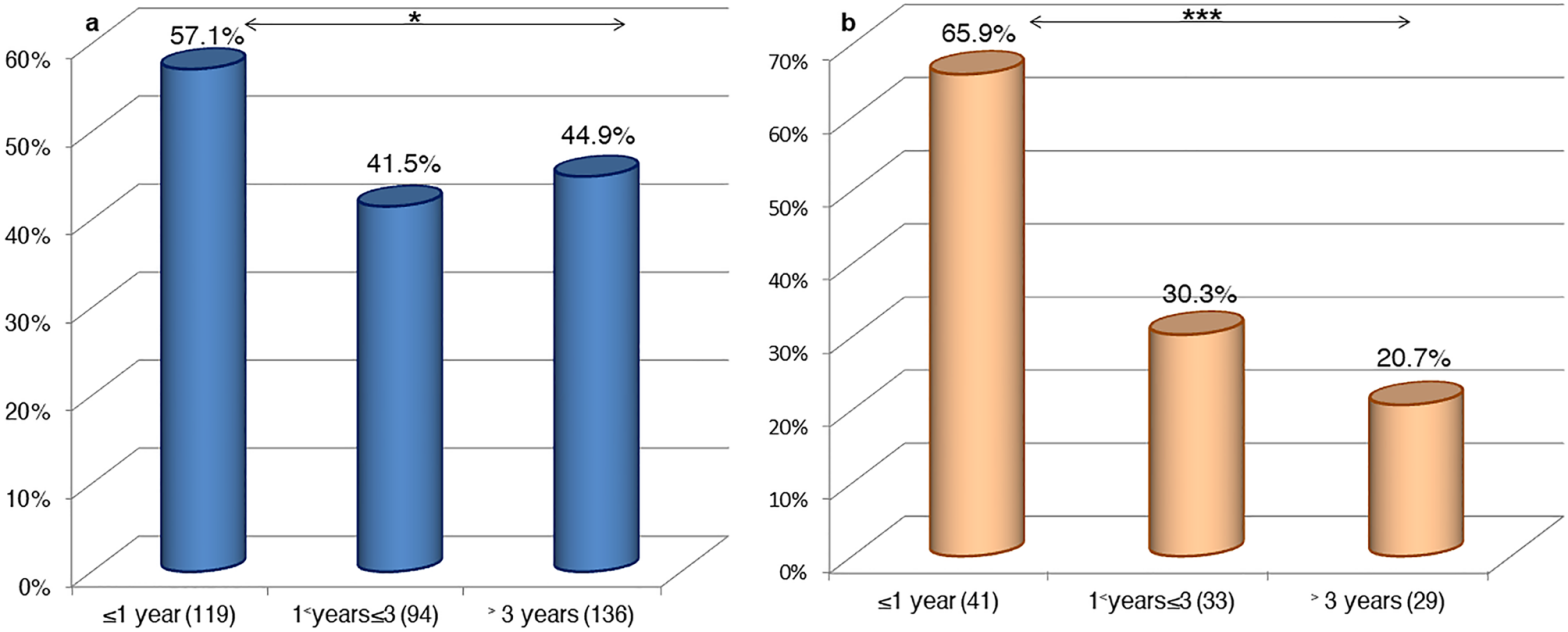
BTV viropositivity rates variation by age in sheep and goats during March 2018–July 2021 in Morocco. (**a**) In sheep. (**b**) In goats (in brackets, the number of samples screened). The statistical significance between rates is indicated as *P < 0.05 or ***P < 0.001.

**Figure 5:**
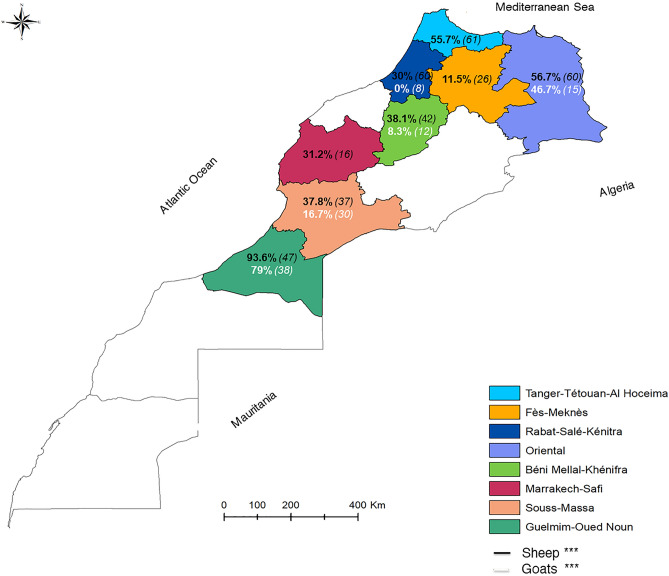
BTV viropositivity rates in sheep and goats during March 2018–July 2021 in Morocco. Black and white colors indicate BTV viropositivity rates in sheep and goats, respectively (in brackets, the number of samples screened). The statistical significance between rates is indicated as ***P < 0.001.

The BTV viropositivity rates differed significantly (χ^2^ = 6.117, P < 0.05) by age in sheep (Fig. 4a). Indeed, the younger animals aged 1 year and less had the highest rate (**57.1%**; 68/119) compared to sheep aged more than 3 years (**44.9%**; 61/136) and those between 1 and 3 years (**41.5%**; 39/94). A significant difference (χ^2^ = 69.05, P < 0.001) in viropositivity rates was also observed between the geographic origin of tested animals; the Guelmim-Oued Noun region had the highest positive rate of **93.6%** (44/47) (Fig. 5).

However, the BTV was slightly more detected in females than males, with viropositivity rates of **50.2%** (115/229) and **44.2%** (53/120), respectively (χ^2^ = 1.155, P ˃ 0.05).

Likewise, the positive animals to BTV were detected in almost similar proportions in Timahdite, Sardi, and crossbreed sheep breeds (**49.3%** (71/144), **47.1%** (66/140), and **47.7%** (31/65), respectively) (χ^2^ = 0.1393, P ˃ 0.05).

### In goats

The RT-qPCR viropositive rates varied significantly with the age, sex, and region of origin of the goats tested (P < 0.05). The Guelmim-Oued Noun region had significantly the highest BTV viropositivity rate in goats (**79%**; 30/38) (χ^2^ = 40.78, P < 0.001) (Fig. 5).

The results also indicated that the BTV viropositivity rate decreased significantly with age (χ^2^ = 16.86, P < 0.001); the younger goats showed the highest rates compared to the older ones (Fig. 4b). Further, the percentage of BTV positives was significantly higher in males (**60%**; 24/40) than in females (**30.2%**; 19/63) (χ^2^ = 8.959, P < 0.01). The indigenous breed of goats was remarkably positive for BTV (**41.8%**; 43/103).

### Variation in BTV viropositivity rate in Moroccan flocks

The extensive breeding had more RT-PCR-positive animals (**51.6%**; 176/341) than the intensive one (**31.5%**; 35/111) (χ^2^ = 13.57, P < 0.001). Furthermore, the percentage of BTV-positive sheep was higher in mixed farms (**53.3%**; 96/180) than in flocks exclusively with sheep (**42.6%**; 72/169) (χ^2^ = 4.02, P < 0.05).

### Variation in BTV viropositivity rate by year and month

**Figure 6:**
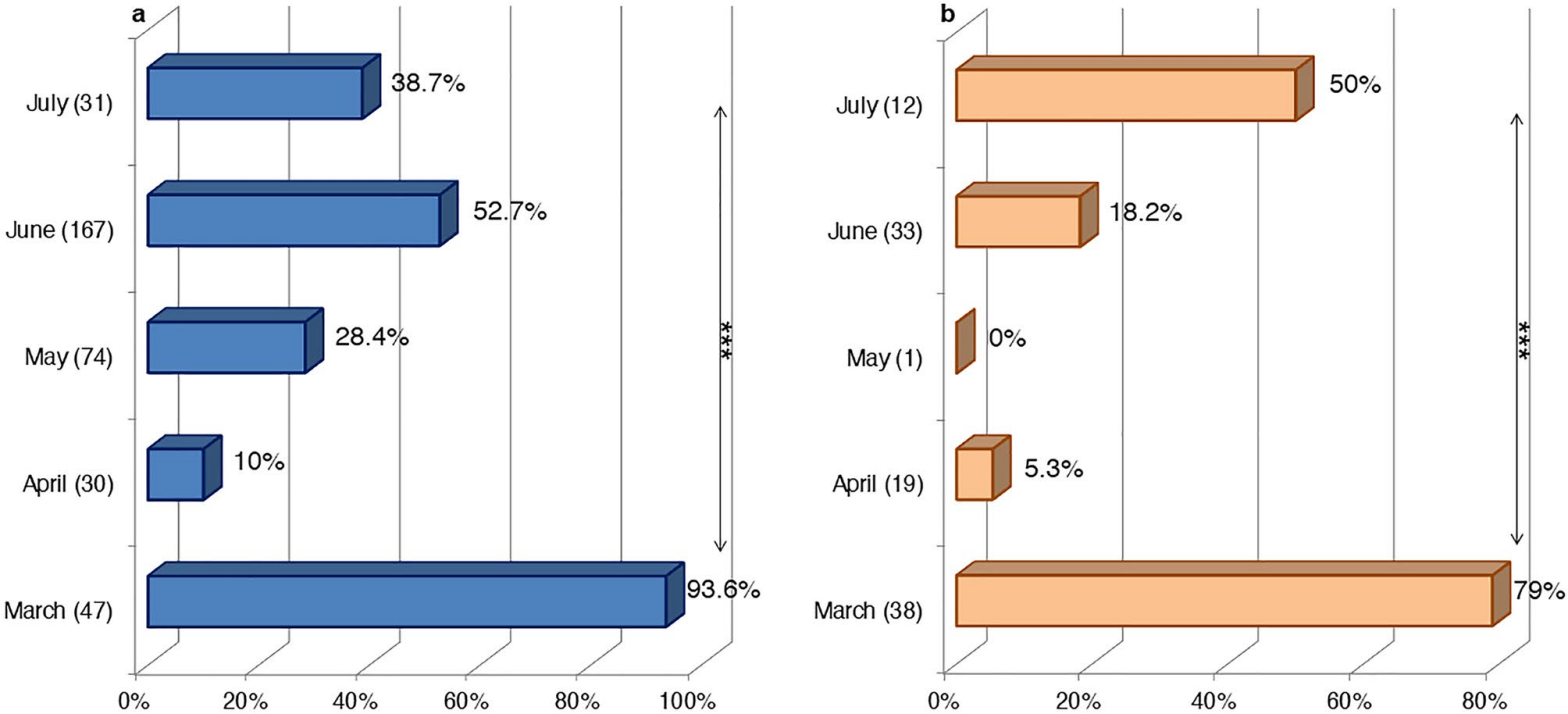
BTV viropositivity rates variation by month of sampling in sheep and goats during March 2018–July 2021 in Morocco. (**a**) In sheep. (**b**) In goats (in brackets, the number of samples screened). The statistical significance between rates is indicated as ***P < 0.001.

The overall BTV viropositivity rate fluctuated significantly over the years of the study (χ^2^ = 24.46, P < 0.001). It varied from **33.8%** (68/201) in 2018, **58.3%** (98/168) in 2019, **50%** (4/8) in 2020 to **54.7%** (41/75) in 2021. On the other hand, the small ruminant samples collected in March had the highest BTV viropositivity rates (**93.6%** (44/47), **79%** (30/ 38), and **79%** (15/19), respectively) (Fig. 6a,b). The statistical variation was highly significant in viropositivity rates according to the month of sampling in sheep (χ^2^ = 70.48, P < 0.001) and goats (χ^2^ = 40.61, P < 0.001).

## Discussion

Understanding and monitoring the epidemiological status of bluetongue disease in an endemic country as Morocco is necessary to implement appropriate measures to control the spread of this disease and its severe socioeconomic consequences.

The current study covers serological and molecular investigations of bluetongue carried out in small ruminants in several Moroccan regions between 2018 and 2021. Serological screening detects BTV group-specific antibodies to assess the nationwide seroprevalence of BT. However, molecular analysis by real-time RT-PCR is necessary to confirm the possible active circulation of the BTV.

According to the findings of the present study, the overall seroprevalence between 2018 and 2021 was 41.7%. This seroprevalence has evolved in comparison to the average seropositivity of 27.68% reported in Morocco between 2008 and 2012 by a previous study^24^ carried out in several regions of the country on suspected sheep with bluetongue. In contrast, this seropositivity was significantly higher in clinical outbreaks reported in sheep (72.31% on average) between 2004 and 2007^24^.

On the other hand, the relatively high RT-PCR positivity rate (46.7%) confirmed the active circulation of BTV in the country. The negative RT-PCR results in seropositive animals can be explained by the fact that these animals have been seroconverted following a previous infection. This rate was lower than that reported in a survey conducted in several regions of Morocco between 2007 and 2012 on sick sheep with clinical BTV symptoms (77.61%)^24^.

The findings can be attributed to the new vaccination strategy of sheep against bluetongue which became voluntary in 2015 and has been limited to the affected farms, resulting in poor vaccine coverage. While previously, vaccination was free of charge and was carried out annually in areas at risk. The use of live attenuated vaccines and the coexistence of several virulent strains silently circulating also trigger the emergence of new reassortant viruses and complicate prevention actions. As well, the emergence of the Foot and Mouth Disease epizootic in 2015 occupied all the vaccination efforts of the authorities that focused on eradicating the new pathogen.

Based on the survey year, the BTV seroprevalence was almost similar in 2018, 2019, and 2021 but increased in 2020 (P ˃ 0.05). However, the seropositivity rate was significantly higher in 2019, 2020, and 2021 compared to 2018. These results were in agreement with the National Veterinary Services (NVS), in which only fewer outbreaks (206 cases notified) were reported in 2018 compared to 2019, 2020, and 2021 (400 cases, 591 cases, and 416 cases notified, respectively)^25^. This can be assigned to the breeding systems adopted and the spatial and climatic features of each region surveyed each year (given that, as mentioned before, different regions were investigated over the four years).

BTV infection is widespread in different countries worldwide with variable seroprevalence rates, including Eastern Sudan (91.2%)^26^, Grenada (78.4%)^27^, Brazil (64.81%)^28^, southwestern Ethiopia (30.6%)^29^, Italy (19%)^30^, Kosovo (11.6%)^31^, Algeria (16.44%)^32^, and China (6.79%)^33^. Likewise, different RT-PCR studies revealed BTV viropositivity of 72.88% in Kosovo^31^, 33.6% in Zambia^34^, and 20.61% in India^35^. The difference in rates reported in various studies could be due to spatial and temporal variations, differences in sample size and sampling techniques, season of sample collection, interaction of *Culicoides* vectors, the vaccination coverage, and the genetic evolution of the BTV.

The results of the current study revealed a statistically significant difference according to the geographical origin of the animals studied. It showed that bluetongue is present throughout Morocco, between the low and medium altitudes. The BTV altitudinal distribution is correlated to the adaptation of the *Culicoides* species to the altitude conditions, particularly the temperature. Some species, such as *C. imicola*, are found in great abundance at low altitudes with temperature conditions favorable for BTV replication^36^.

In sheep, all farms from the eight surveyed regions had seropositive animals. This is evidence of active BTV circulation. The regions presenting the highest rates of BTV infection were the Guelmim-Oued Noun, Béni Mellal-Khénifra, Souss-Massa, Oriental, and Tanger-Tétouan-Al Hoceima regions.

Guelmim-Oued Noun registered the highest seropositivity (87.3%) and BTV viropositivity rate (93.6%).The region is located on the southern coast of Morocco and is considered the Atlantic gateway to the Sahara (desert). The breeding system adopted in this region focuses on nomadism. The animals are constantly on the move and are subject to the stress of transit, other concurrent infections, and other predisposing factors that may weaken individual or herd immunity and therefore increase the susceptibility of the animals to be infected.

Béni Mellal-Khénifra had similarly significant seropositivity (63.5%) and viropositivity rates (38.1%). This region has a potential for animal production covering all species in Morocco. Livestock farming of rustic breeds adapted to the local context is predominant. The breeding systems practiced are the extensive type, constituting the main occupation and source of income for the rural population. The animals are always on the grazing land and therefore in contact with the vector. Most *Culicoides* species are in activity around dusk and at night, but many *Culicoides* species are active through the day, with two peak biting times: after sunrise and near sunset. These feeding times can be extended during low light and overcast circumstances, resulting in biting all day^37^. However, the high seroprevalence in this region compared to the RT-PCR positivity rate could indicate an ancient circulation of the bluetongue virus.

Souss-Massa had a remarkable rate of seropositivity (50.7%) and BTV viropositivity (37.8%). The region is a junction between the north and the south of the country. It is bordered in the southeast by Algeria, the southwest by the Guelmim-Oued Noun region, and the west by the Atlantic Ocean. The results achieved can be explained by the geo-climatic characteristics of this region and the transhumant movement of herds coming from endemic southern areas. Indeed, in our study, extensive farms had the highest seroprevalence and RT-PCR positivity rates compared to intensive farms. The animals are more exposed to *Culicoides* vector bites and the risk of BTV infection in the pasture than in the stall^38^. And based on the results of a study in China^39^, the intensive farms had more advanced technologies to reduce bluetongue vectors and infections.

The Oriental region, in northeastern Morocco near the Moroccan border, exhibited elevated BTV infection rates (a BTV seropositivity and viropositivity rate of 40.8% and 56.7%, respectively). Their geography presents a gateway for the entry or exchange of infected vectors. Also, their ecological conditions foster the development of the vector population and the occurrence of the disease.

Tanger-Tétouan-Al Hoceima region also had high BTV infection rates (a BTV seropositivity and viropositivity rate of 34.8% and 55.7%, respectively). It is situated in northwest Morocco, bordered on the west by the Atlantic Ocean and the east by the Mediterranean Sea. The region is rich in hydrological resources (Oued Loukkos, etc.). The surroundings are particularly agricultural due to the rivers and a large proportion of plantations. These factors create a favorable environment for the *Culicoides* multiplication.

These observations on the influence of geography origin on sheep results can also be applied to goats, where the highest BTV rates were registered in Guelmim-Oued Noun and the Oriental region. This region impact was also observed in previous studies^39,40^, where it was indicated that the transmission dynamics of BTV are significantly reliant on the region and its characteristics.

The survey was conducted between March and July (2018–2021). During this period, there was some variance in infection rates. March had a high viral circulation in sheep and goats. This month marks the transition from winter to spring. It is characterized in Morocco by average temperatures ranging from 12 to 24 °C depending on the region. These climatic conditions can be favorable for certain *Culicoides* species and not for others. Generally, the seasonal abundance of *Culicoides* differs between species showing three distinct peaks in spring, summer, and autumn. Some Palearctic *Culicoides* vectors, such as *Culicoides scoticus*, are generally at their highest abundance in the first or last months of the year. *C. newsteadi*, a very abundant species in Morocco, marks a high level of abundance between February and May. Because of their winter-spring activity period, *C. newsteadi* species are the potential candidates for viral overwintering^41^. Based on a research study in Northern Europe^42^, *Culicoides* infected early in the winter would be able to overwinter and restart BTV transmission 90–120 days or more later. As a result, the limited population of *Culicoides* active in winter would be sufficient to maintain the virus circulation. It also demonstrated in Sardinian study^41^ that pools of *C. obsoletus* and *C. newsteadi* were BTV positive for catches during the winter-spring months (December–May) and infrequently in summer (July). On the other hand, other species present abundance peaks in late spring, summer, and fall^43^. The variation in BTV infection rates observed in the current investigation could be related to the climate conditions for every region at the time of sampling. As a country in the Mediterranean region, Morocco is potentially vulnerable to climate changes, the environmental impacts of which are likely to be wide and varied in the country. Hence, the prevalence of the disease and its geographic and seasonal repartition could also change, as it is closely related to climate and environmental changes. Indeed, these changes impact the life cycle of the vector and its vectorial capacity and competence. As for *Culicoides imicola*, climate change could expand its range in latitude and altitude and increased its reproductive frequency with more generations per year^44^. Likewise, climate change could impact the viremia duration within the host that can last longer than 60 days^45^, particularly in reservoir species like cattle^42^.

The no significant difference (P ˃ 0.05) in seroprevalence and viro-positivity rates between sheep and goats indicates that both species are likely to be exposed to the same risk of BTV infection. Sheep are very susceptible to BTV with noticeable clinical signs^27^. However, goats are usually asymptomatic or subclinically infected. The active BTV circulation among goats suggests that this species plays an important role in disease epidemiology and can serve as a source of infection to other susceptible animals^26,46^. A serological survey conducted in 2005 also illustrated the role of goats in BTV incidence in Morocco. BTV seropositivity was high in goats (79% in the declared infected area and 32% in the non-infected area). Seropositivity in sheep was lower, at 59% in the declared infected area and 15% in the other regions^47^. The involvement of goats was also observed in a study conducted in Zambia, where the BTV prevalence in goats was significant^34^. Comparable serological results were recorded in 2016 in Egypt^48^ (sheep: 41.86%, goats: 24%) and Grenada^27^ (sheep: 71.7%, goats: 80.2%).

This discrepancy observed between species is presumably due to their different immune responses to BTV infection^49^ and the fact that there is no vaccination policy against bluetongue in goats in Morocco.

During BTV infection, the ratio of thromboxane/prostacyclin (inflammatory and vasoactive mediators) in sheep is greater than in other species. This difference may explain the increased susceptibility of sheep to vascular injury and thrombosis and the unequal intensity of clinical signs between species^50^. Natural infection usually results in prolonged bluetongue viremia in many ruminants due to the interaction of the virus with erythrocytes and other blood cell types^51,52^. As well, in sheep, peripheral blood mononuclear cells (PBMCs) sustain higher levels of viral replication than goats^49^. Viral RNA can be detected up to 222 days post-infection by RT-qPCR^53^. Alternatively, BTV could persist in some lymphocytes from sheep (e.g. *γδ*T-cells activated by skin inflammation induced by infected *Culicoides* bites). The virus escapes from infected lymphocytes by budding across the cell membrane. The BTV outer capsid proteins would be covered by a lymphocyte membrane, making them less susceptible to antibodies. Therefore, enveloped viral particles may be capable of binding to other lymphocytes. The direct connections between infected and uninfected lymphocytes could lead to the persistence and transmission of BTV^54^. It is also documented that goats retain high viral titers of BTV antigen^46^, and they involve a wide range of immune genes in immune system processes^49^.

Indeed, in the current study, the mixed farms combining sheep with goats and eventually cattle presented increased sheep infection rates than those exclusively of sheep. That implies that goats are potential BTV reservoirs, highlighting their relevant contribution to the persistence of the BTV in Morocco.

The animals screened in this survey did not show BT symptoms, suggesting a silent circulation of the bluetongue in Morocco besides the annual declarations of the disease. As pointed in Malawi^55^, the absence of clinical manifestation indicates that indigenous animals have a high degree of innate resistance. Generally, the natural infection also provides long-term homologous protection. The animal is clinically protected against the same serotype that it is infected with^56^. This notwithstanding, the proportion of immunized animals decreases yearly with the birth of youngsters and the renewal of the breeding herd. The silent spread of multiple serotypes in Moroccan farms can contribute to new variants and, consequently, to severe epidemics.

The present study found a significant connection between BTV infection rates and the age of the animals examined. Older sheep had progressively higher seroprevalences than younger animals. However, the BTV was more active in sheep aged 1 year or less and those more than 3 years. These findings could confirm the continued infection in Morocco caused by recurrent exposure of older animals to infected *Culicoides*. The RT-qPCR results suggest that young animals become susceptible to BTV infection after 6 months of age, and this may be secondary to the disappearance of protective maternal antibodies^57^. The active circulation of BTV in sheep above 3 years of age strongly confirms recent BTV reinfection. In goats, the BTV seropositivity and viropositivity rates decreased with age. Serological results were non-significant and may hypothesize a limited persistence of antibodies against BTV in goats with a difference in immune response depending on the serotype involved. In a study in Liechtenstein^58^, it was observed that the infection of goats with BTV-25 induced a limited activation of the immune system resulting in a weak immune response against BTV VP7. The BTV viropositivity in young goats indicated that the BTV was circulating during the survey period^59^.

Females had higher rates (ELISA and RT-qPCR) than males in sheep. The RT-qPCR result was not statistically significant (P ˃ 0.05). That implies that both sexes are at the same risk of infection. The fluctuation in seroprevalence rates can be ascribed to husbandry practices in Morocco. Male sheep are typically graded for fattening and therefore are protected from the bites of *Culicoides*. As well, they are frequently slated for sale and slaughter at a young age, resulting in a higher proportion of older females than older males. Nonetheless, females are usually kept for reproduction and are thus regularly exposed to several BTV-infected vector episodes. Likewise, they are generally physiologically active during mating and lactation and are likely to be more vulnerable to BTV infection. The difference could also be due to the inherent susceptibility of females to BTV infection or the influence of other female-specific factors^57^. As it was previously observed for other diseases, females may have a greater number of immune genes and cells that promote a better immune response and high antibody production (quantity and quality)^60^. However, the BTV infection rates were more significant in male goats than females. This might be explained by the fact that males are more exposed to *Culicoides* bites because they occupy different and extensive home ranges due to their behavior and wider use of space, while the females live in small stable groups^61^. Furthermore, given that male goats have a higher weight than females, we could consider the hypothesis suggesting the possibility of a preference of the vector for animals with greater body mass^62^. Other studies^63,64^ have similarly reported an association of BTV infection with age and sex factors.

Antibodies against BTV were also highly detected in the sheep breeds studied, with a significant percentage in the Timahdite and Sardi breeds. RT-qPCR proved a considerable BTV circulation with similar non-statistically significant proportions. These high levels accord with the findings of an earlier study in Morocco^47^, which suggest that these two breeds are more susceptible to BTV infection than others in the country. Indeed, after the BTV serotype 1 epidemic recorded in Morocco in 2006, the sheep of the Middle Atlas region, where the Timahdite breed dominates, were the most affected with the highest morbidity and mortality rates and then the cradle of Sardi breed sheep^47^. Multiple studies have also reported the influence of animal breed on the bluetongue's seroprevalence, clinical form, morbidity, and mortality rates^22,30^. This breed susceptibility to bluetongue was confirmed by further research^65,66^. As per these studies, sheep breeds from subtropical regions, where the disease was endemic, were more resistant, while improved European breeds were more susceptible. These can be explained by the variation in the innate response, the number of over-or under-expressed genes implicated in the cellular immune response, and the timing of the neutralizing antibody response intervention for each breed^65^.

## Conclusion

This study is the first in Morocco to assess the BTV infection rates and risk factors of its transmission among small ruminant herds. The current serological and molecular investigations revealed that the BTV is endemic throughout the country. The epidemiological evolution of bluetongue in Morocco can be explained by the absence of an effective and sustainable preventive vaccination program, the co-existence of reservoirs and susceptible animals, the host response to infection, and the silent circulation of the BTV within Moroccan livestock. The transhumance factor and the limited control of susceptible animal movements promote the BTV circulation throughout the country. Furthermore, the recent extensions of the *Culicoides* biotope, the overwintering mechanisms, and the variations in the seasonality of *Culicoides* species attribute to the persistence of BTV in Morocco. Age, sex, breed, season, geography, and, most importantly, type of breeding are essential risk factors influencing the infection with bluetongue.

Therefore, a more systematic study based on the identification and isolation of different BTV strains circulating in Morocco, a consistent and well-defined control strategy based on vaccination programs, as well as the surveillance of vectors and limitation of animal transport between different regions of the country, are essential to prevent and control the bluetongue.

## Data Availability

All data generated or analyzed during this study are included in this published article.
